# Bioinformatics prediction and experimental verification identify cuproptosis-related lncRNA as prognosis biomarkers of hepatocellular carcinoma

**DOI:** 10.1016/j.bbrep.2023.101502

**Published:** 2023-06-21

**Authors:** Zhu Liangyu, Zhang Bochao, Yin Guoquan, Zhang Yuan, Li Heng, Zhou Hanyu

**Affiliations:** a Central Laboratory, The First Affiliated Hospital of Wannan Medical College, Wuhu, Anhui, China; b Pasteurien College, Suzhou Medical College, Soochow University, Suzhou, Jiangsu, China

**Keywords:** Cuproptosis, Hepatocellular carcinoma, CAlncRNAs, Prognostic model, TCGA

## Abstract

Cuproptosis is a form of cell death caused by intracellular copper excess, which plays an important regulatory role in the development and progression of cancers, including hepatocellular carcinoma (HCC), a prevalent malignancy with high morbidity and mortality.

This study aimed to create a cuproptosis associated long non-coding RNAs (CAlncRNAs)signature to predict HCC patient survival and immunotherapy response. Firstly, we identified 509 CAlncRNAs using Pearson correlation analysis in The Cancer Genome Atlas (TCGA) datasets, before the three CAlncRNAs (MKLN1-AS, FOXD2-AS1, LINC02870) with the most prognostic value were further screened. Then, we constructed a prognostic risk model for HCCwas using univariate and LASSO Cox regression analyses. Multivariate Cox regression analyses illustrated that this model was an independent prognostic factor for overall survival (OS) prediction, outperforming traditional clinicopathological factors. And the risk score not only could be prognostic factors independent of other factors but also suited for patients with diverse ages, stages, and grades. The 1-, 3-, and 5- years areas under the curves (AUC) values of the model were 0.759, 0.668 and 0.674 respectively. Pathway analyses showed that the high-risk groupenriched in immune-related pathways. Importantly, patients with higher risk scores exhibited higher mutation frequency, higher TMB scores, and lower TIDE scores. Besides, we screened for two chemical drugs (A-443654 and Pyrimethamine) with the greatest value for high-risk HCC patients. Finally, the abnormal high expression of the three CAlncRNAs were confirmed in HCC tissues and cells by Real Time Quantitative PCR (RT-qPCR). And proliferative, migratory and invasion abilities of HCC cell were restrained via silencing CAlncRNAs expression *in vitro.*

In summary, we built a CAlncRNAs-based risk score model, which can be a candidate for HCC patients prognostic prediction and offer some useful information for immunotherapies.

## Highlights

• This study aimed to create a novel signature to predict patient survival and immunotherapy response in patients with HCC.
• The CAlncRNAs-based risk score model can be employed as a candidate for prognostic prediction of HCC patients and has been tentatively shown to have an important role in HCC immunotherapy.

## 1 Introduction

Primary liver cancer is the sixth most prevalent malignancy and the fourth leading cause of cancer mortality worldwide, posing a serious threat to human health [1]. HCC accounts for about 75%–85% with a five-year overall survival rate (OSR) of only 10%–18%, as most HCC patients are diagnosed at an advanced stage, losing the opportunity for surgery and having a poor prognosis [1]. Nowadays, the main treatment for HCC is surgical resection, but the treatment options for unresectable patients with middle to advanced HCC are very limited, and therefore new treatment strategies are urgently needed. Although immunotherapy has revolutionized cancers including HCC treatment, still faces poor surgical outcomes [2,3]. In addition, high postoperative of metastasis and recurrence severely affects the outcome and survival of HCC patients [4]. Therefore, exploring new effective prognostic marker models and therapeutic approaches is urgently required to improve HCC patient survival.

Recently, a new intracellular copper ion-dependent and regulated mode of cell death have been identified: copper toxicity [5]. Copper ion binds directly to the mitochondrial lipoylated components of the tricarboxylic acid cycle pathway, resulting in abnormal aggregation of mitochondrial lipoylated proteins and loss of Fe–S cluster proteins, leading to a protein toxic stress reaction and eventually cell death. Copper toxicity occurs by mechanisms different from all other mechanisms known to regulate cell death, including apoptosis, ferroptosis, pyroptosis, and necroptosis [6]. To identify specific metabolic pathways for cuproptosis, seven positively and three negatively regulated genes were identified, of which FDX1 and Protein lipoylation are key regulators of cuproptosis [5]. The liver, as the most important organ in metabolic system, is closely associated with copper enrichment, especially in cancer cells with higher demand for copper [[7], [8], [9]]. Several prospective cohort studies have shown that higher level of copper in serum was strongly associated with poorer HCC prognosis and could be a useful predictive marker for survival in HCC cases [[10], [11], [12]]. In addition, Sawaki et al. [13] reported that accumulation of copper may promote the development of HCC in LEC rats by fostering a favorable growth environment. However, the relationship between cuproptosis with HCC progression and immune response has not been reported. Therefore, it is crucial to comprehend the probable mechanisms of cuproptosis-related gene alterations in HCC as well as their implications for immunotherapy and prognosis. Until now, only 19 coding genes verified to be related to cuproptosis, which was insufficient for HCC prognostic model building. The ability of long non-coding RNA (lncRNA) to interact with coding genes and play a regulatory role in cancer gives us a great idea.

LncRNA is an important component of non-coding RNA, involved in numerous diseases processes *in vivo*, especially cancer [[14], [15], [16]]. Researches showed that lncRNAs, as oncogene [17,18] or suppressor [19], play an important role in regulating the progression, metastasis, and invasion of HCC. Lnc-ZEB1-AS1 enhances the expression of ZEB1 by elevating its promoter activity thus promoting the metastasis of HCC, and patients with ZEB1-AS1 hypomethylation are anticipated to have a high metastatic recurrence and poor survival outcomes [20]. In addition, LncRNA can also serve as an important marker for prognostic prediction of HCC. Marwa et al. [21] found that lnc-WRAP53 was a significant independent prognostic marker in relapse-free survival of HCC patients. Lnc-ATB, which is involved in tumor metastasis, was upregulated in HCC metastasis and predicted poor prognosis in HCC patients [22]. In contrast, lnc-miR503HG with metastasis suppression function predicted a good prognosis [23]. Nevertheless, CAlncRNAs have not been considered HCC prognostic indicators or possible therapeutic targets. Although a large number of modules have been constructed using lncRNAs to predict survival outcomes in HCC, no CAlncRNAs prediction model has been attempted. Consequently, it is necessary to explore CAlncRNAs, which might provide new ideas and insights for HCC prediction and treatment.

In this study, we constructed a novel predictive modle based on three CAlncRNAs (MKLN1-AS, FOXD2-AS1, LINC02870) to forecast the prognosis of HCC and immunotherapy. Our results suggested that these CAlncRNAs can be considered as prognostic biomarkers for HCC, providing some theoretical references for postoperative survival prediction and molecularly targeted precision therapy and elucidating the pathogenesis of cuproptosis in HCC. Finally, we performed *in vitro* experiments to validate the expression and functions of CAlncRNAs in HCC.

## 2 Materials and methods

### 2.1 HCC data acquisition and preprocessing

**Fig. 1:**
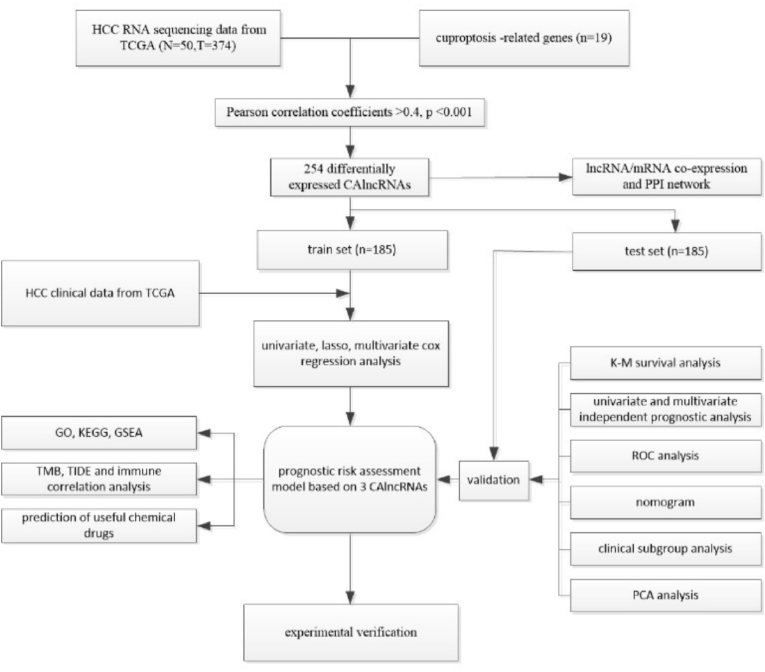
Study flowchart showing the process of constructing the 3-CAlncRNAs model to predict prognosis of HCC.

The study protocol is shown in Fig. 1. The transcriptomic data and clinical data of HCC patients in TCGA were downloaded and collated from the GDC database (https://portal.gdc.cancer.gov/) in the present study. A total of 374 tumor datasets and 50 normal datasets were obtained (had been normalized to Fragments Per Kilobase Million (FPKM) format) with the corresponding clinical data (OS, Survival state, Age, Gender, Grade, Stage, Tumor-Node-Metastasis (TNM) stage). To reduce bias, the HCC patients with missing OS values or short OS values were removed (<30 days). Totally 19 cuproptosis-related genes (NFE2L2, NLRP3, ATP7B, ATP7A, SLC31A1, FDX1, LIAS, LIPT1, LIPT2, DLD, DLAT, PDHA1, PDHB, MTF1, GLS, CDKN2A, DBT, GCSH, DLST) were retrieved from previous literature [5]. The samples were randomly divided into the train and test sets by Strawberry Perl and “caret” R package of R software (version: 4.1.0). Chi-square test were employed to determine the differences in clinical characteristics (age, sex, and TNM stage) between the two sets.

### 2.2 Identification of differentially expressed CAlncRNAs

The Homo_sapiens.GRCh38.95.chr.gtf.gz file was downloaded for gene id conversion from the Ensembl database. The matrix of gene symbol numbered expression was processed with the filter of the lncRNA expression data and mRNA expression data. The “limma” R package was applied to screen the pre-processed lncRNA expression data to derive differentially expressed lncRNAs (|Log_2_ fold change (FC)| > 1, false discovery rate (FDR) < 0.05). Heatmap and volcano map of differentially expressed lncRNAs were drawed by the “heatmap” R package.

The train set of HCC mRNA expression data was intersected with 19 cuproptosis-related genes to obtain the cuproptosis-related genes expressed in HCC tissues. The “limma” R package was applied for the co-expression analysis of differentially expressed lncRNAs with cuproptosis-related genes expressed in HCC tissues, resulting in differentially expressed CAlncRNAs (Pearson correlation coefficients >0.4, p < 0.001).

The “igraph” R package was applicated to demonstrate the mutually regulated connection between CAlncRNAs and corresponding target mRNAs. The sankey relational diagram was performed by “ggplot2” R package. And the protein-protein interaction (PPI) network of 19 cuproptosis-related genes was constructed by STRING (https://string-db.org/) website.

### 2.3 Construction and verification of the prognostic gene model

First, the prognostic-classified lncRNAs were selected by using univariate cox regression with p value < 0.05. After that, we screened for CAlncRNAs with 10-fold cross-validation based on LASSO regression analysis. The screened CAlncRNAs were then selected for multivariate cox proportional hazards regression and risk model construction. Finally, the screened gene expression was multiplied by the multivariate Cox regression coefficient to construct a prognostic risk model with calculated risk score. Meanwhile, the optimal model was selected based on the Akaike information criterion (AIC) value. The formula is as follows: **Risk score=Coef*(lncRNA1)*×Exp*(lncRNA1)*+Coef*(lncRNA2* × Exp*(lncRNA2)*+……+Coef*(lncRNAn)*×Exp*(lncRNAn)***, where Exp*(lncRNAn)* represents the expression level of a particular lncRNA and Coef*(lncRNAn)* represents the lncRNA coefficient in multivariate Cox regression analysis.

Subsequently, we divided the patients of dataset into two groups as high risk and low risk based on the median risk score. The “tidyverse”, “ggplot2” and “ggExtra” R packages were used to plot the correlation heatmap of the 19 cuproptosis-related genes with the CAlncRNAs of model. The K-M analysis and progression-free survival (PFS) curves were plotted using the “survivor” and “survminer” R packages based on log-rank test. The “pheatmap” R package were enrolled for plots risk profiles maps, survival status maps, and risk heatmaps. Then, univariate analysis and multivariate Cox regression analysis were employed to determine the association between the risk scores and clinical characteristics. Receiver operating characteristic (ROC) curves, and the AUC were presented by“survival” and “survminer” packages. Finally C-index curves were plotted by the “dplyr”, “survival”, “rms” and “pec” R packages to compare the difference in prognostic accuracy between risk scores and clinical characteristics.

### 2.4 Establishment of nomogram

With the “rms” R package, we established nomogram based on independent prognostic factors to predict 1-, 3-, and 5-year OSR of HCC patients. We also plotted calibration curves based on the Hosmer-Lemeshow test to determine uniformity between the predicted results of nomogram and the actual 1-, 3-, and 5-year OS probabilities.

### 2.5 PCA analysis

PCA was performed on whole gene data (mRNA and IncRNA), cuproptosis-related mRNA data, CAlncRNAs data, and IncRNA data of prognostic models using the “scatterplot3d” and “limma” R packages, and the corresponding scatterplot was plotted separately by downscaling and visualizing.

### 2.6 Exploration of pathway enrichment and molecular mechanisms

The “clusterProfiler” R package was used to predict their functions based on Gene Ontology (GO) enrichment analysis and Kyoto Encyclopedia of Genes and Genomes (KEGG) pathway enrichment analysis. P < 0.05 was considered as significant correlation of gene function according to GO database and KEGG pathway. Genomic enrichment analysis (GSEA) was performed using GSEAv4.2.2 software with an absolute value of normalized enrichment score (|NSE|) > 1.

### 2.7 TMB, TIDE and immune correlation analysis

First, the somatic mutation data (TCGA.BRCA.varscan.DR-10.0.somatic) was downloaded from TCGA. Then the “maftools”, “survival” and “survminer” R packages were used to integrate the data and analyze the diverse survival of TMB between high and low risk group. The TIDE score between two groups was downloaded from online database (http://tide.dfci.harvard.edu/).

The immunocyte infiltration analysis was performed between high and low risk groups according to the Tumor Immune Estimation Resource (TIMER) 2.0 (http://timer.cistrome.org/), with the infiltration fraction calculated in TME via Wilcoxon signed-rank test, “limma”, “scales”, “ggplot2” and “ggtext” R packages.

To explore the Potential immune checkpoint, we compared 47 immune checkpoint (Table.s.1) related gene expression between high and low risk groups by “limma”, “reshape2”, “ggplot2” and “ggpubr” R packages.

In addition, immune-related function heatmap was performed according to the immune function set file (Table.s.1) by “limma”, “GSVA”, “GSEABase”, “pheatmap” and “reshape2” R packages between the two groups.

### 2.8 Prediction of useful chemical drugs

To discovere the useful chemical drugs used in LUAD therapy, we compared the half maximum inhibitory concentration (IC50) values of the drugs obtained from the GDSC website (https://www.cancerrxgene.org/). The results were visualized by the “pRRophetic”, “limma”, “ggpub”, and “ggplot2” R packages.

### 2.9 Cell cultures and RT-qPCR

Human hepatocytes L-02 cells and HCC cells (Hep2G and Hep3B) were purchased from the Institute of Cell Biology, Chinese Academy of Sciences (China). All cells were cultured in Dulbecco's modified Eagle's medium (DMEM)-HEPES medium supplemented with 10% foetal bovine serum (FBS, Gibco) at 37 °C and 5% CO_2_. Thirteen matched tumor and adjacent non-tumor tissues were obtained from HCC patients after excluding tissues with significant inflammatory infiltrate, liver fibrosis, steatosis and other non-tumorigenic lesions.

Total RAN was isolated using the RNeasy kit (QIAGEN), and cDNA was synthesized using the iScript cDNA synthesis kit. RT-qPCR was performed with SYBR Green PCR Master Mix and CFX96 real-time PCR system. Calculations of CAlncRNAs expression levels were performed using the comparative CT (ΔΔCT) method and normalized against GAPDH snRNA levels. All PCR tests were run in triplicate. The primer sequences used are shown in Table 1.

**Table 1:** PCR primer sequences.

Gene	Primer sequences(5′–3′)
MKLN1-AS (F)	AAAGAGTATGTCGCTTATTGTCTAAGA
MKLN1-AS (R)	ATCCTGCTGACTTACTCCAGATGT
FOXD2-AS1 (F)	TGGACCTAGCTGCAGCTCCA
FOXD2-AS1 (R)	AGTTGAAGGTGCACACACTG
LINC02870 (F)	AGGCAGCTCCCGTGGTAGAT
LINC02870 (R)	GAAGGCCGCATTCCTGAAAC
GAPDH (F)	GGTTGTCTCCTGCGACTTCA
GAPDH (R)	TGGTCCAGGGTTTCTTACTCC

**Note:** F, forward; R, reverse.

### 2.10 SiRNAs transfection

MKLN1-AS, FOXD2-AS1 and LINC02870 siRNAs, negative control (NC) were all ordered from HANBIO (China). siRNAs were transfected into HepG2 cells with FECT™ CP Transfection Reagent (RIBOBIO, China) according to the manufacturer’s standard protocol. All of the siRNA sequences are shown in Table 2.

**Table 2:** siRNA sequences.

Gene	Sequences(5′–3′)
MKLN1-AS siRNA (Sence)	UACAUUAACAUAACAUAAGCU
MKLN1-AS siRNA (Antisence)	CUUAUGUUAUGUUAAUGUAAA
FOXD2-AS1 siRNA (Sence)	GCGAAGAGUACGUUGCUAUTT
FOXD2-AS1 siRNA (Antisence)	AUAGCAACGUACUCUUCGCTT
LINC02870 siRNA (Sence)	CGUGGUAGAUCAAGCCUCATT
LINC02870 siRNA (Antisence)	UGAGGCUUGAUCUACCACGTT
NC siRNA (Sence)	UUCUCCGAACGUGUCACGUTT
NC siRNA (Antisence)	ACGUGACACGUUCGGAGAATT

### 2.11 Cell proliferation assay

Cell counting kit‐8 (CCK‐8) HANBIO (China) assay was performed to investigate the proliferation capacity of HepG2 cell. Cells were seeded in 96‐well plates at a density of 4 × 10^3^ cells per well and transfected with siRNAs after cell adherence under normal cell culture conditions. CCK‐8 solution was then added to each well at 24, 48, 72, and 96 h post‐transfection. HepG2 cell viability was analyzed using a microplate reader at 450 nm.

### 2.12 Wound healing assay

After reaching 100% confluence, HepG2 cells were wounded by scraping with a 200 ml tip, following washed three times in PBS and incubated in serum-free medium. Wounds were observed at 0 and 48 h. The wound healing percentage was calculated by the following formula: wound healing percentage = migration distance/distance at 0h.

### 2.13 Invasion assay

The serum-free transfected HepG2 cells suspension was placed on the upper layer of Transwell chamber coated with Matrigel matrix, and the medium with 10% FBS was added. After being incubated for 24 h with 5% CO_2_ at 37 °C, we used a cotton swab to wipe off the cell membrane surface, whereas the invasive cells on the lower surface were fixed and colored with 0.1% crystal violet for half an hour. By observing the cell numbers in lower chamber we were able to quantify cell invasion ability.

### 2.14 Apoptosis

Apoptotic levels of the collected cells were determined using an Annexin V-FITC apoptosis detection kit (eBiolegend, Inc.). Cells were suspended in 100 μl binding buffer and then stained with 10 μl Annexin V-FITC and 5 μl PI at room temperature for 15 min without light. The percentage of early + late apoptotic cells were measured using a flow cytometer (Beckman Coulter, Inc.) and data was analyzed using FlowJo software (Treestar).

### 2.15 Statistical analysis

All data were presented as the $\bar{x}$ ± SD. And all experiments were repeated independently at least three times. Appropriate statistical methods including Student's *t*-test, one-way ANOVA followed by Bonferroni *post-hoc* test were used to calculate differences between groups by GraphPad Prism 8. software. P-values * <0.05 was considered statistically significant difference.

## 3 Results

### 3.1 Identification of differentially expressed CAlncRNAs

**Fig. 2:**
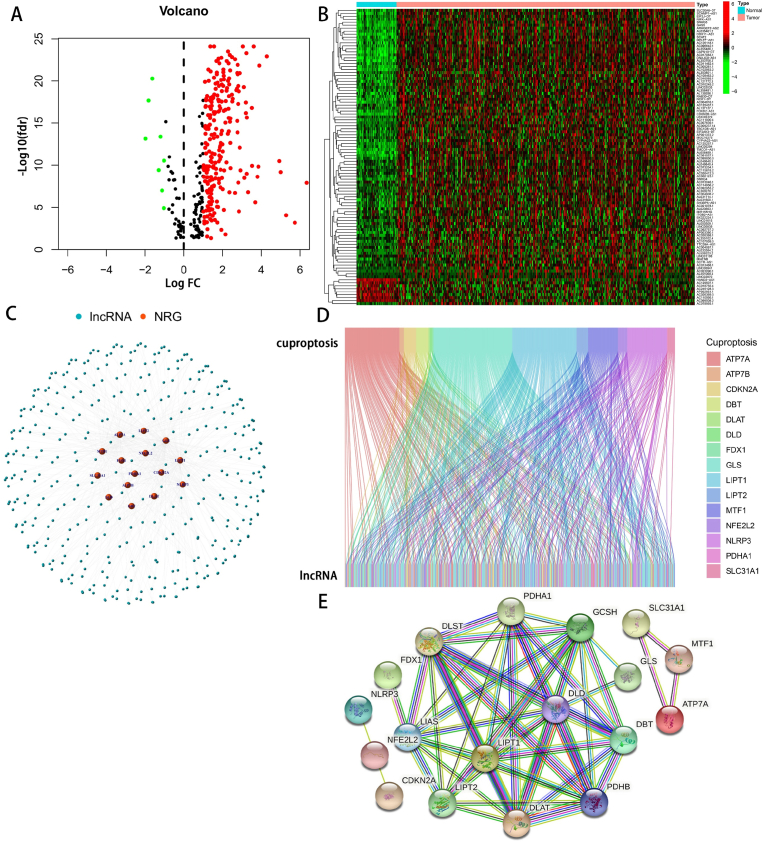
Construction of prognostic cuproptosis-related risk model composed of the three CAlncRNAs. (A) Volcano plot presenting differentially expressed lncRNAs discovered from HCC tissues compared with normal samples from TCGA dataset. (B) Heatmap presenting differentially expressed lncRNAs discovered from HCC tissues compared with normal samples from TCGA dataset. (C) Co-expression relationship between HCC differentially expressed lncRNAs and cuproptosis-related genes. (D) Sankey diagram for the network of cuproptosis-related genes and HCC differentially expressed lncRNAs. (E) PPI network of 19 cuproptosis-related genes.

We downloaded 50 normal samples and 374 HCC samples from The Cancer Genome Atlas (TCGA) matrix. According to the expression of 19 cuproptosis genes, we screened 509 CAlncRNAs with the standard of correlation coefficients >0.4 and P < 0.001. Among the CAlncRNAs, we got 254 differentially expressed CAlncRNAs (|Log_2_FC| > 1 and P < 0.05) between the normal and tumor groups, including eight downregulated lncRNAs and 246 upregulated lncRNAs. All these genes were clustered and analyzed by the relevant volcano and heatmaps (Fig. 2A,B). The potential correlations between the lncRNAs and mRNA were demonstrated by network Fig., sankey relational diagram and PPI.(Fig. 2C–E).

### 3.2 Construction the prognostic risk assessment model with CAlncRNAs

**Fig. 3:**
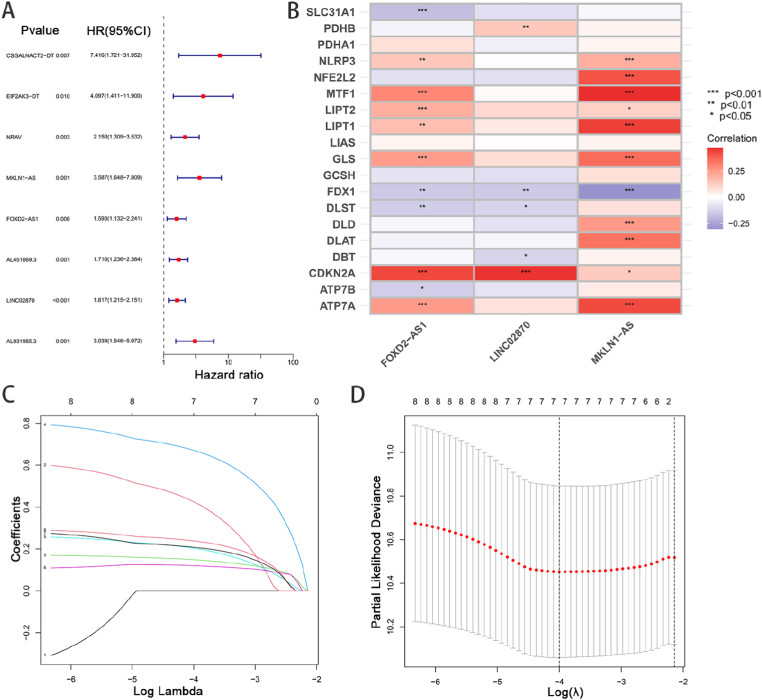
Construction of prognostic cuproptosis-related risk model composed of three CAlncRNAs. (A) Forest plot of the prognostic values of the prognostic model based on eight cuproptosis-related lncRNAs. (B) Correlation heatmap showed the correlation of cuproptosis-related genes with the three lncRNAs of our model. (C) The LASSO coefficient of -related lncRNAs in HCC. (D) Selecting the best parameters for HCC on the basis of LASSO model (λ). ***P < 0.001; **P < 0.01; *P < 0.05.

Using random grouping method, the HCC differentially expressed CAlncRNAs data were equally divided into train and test sets. Combined with clinical information, univariate COX regression analysis was performed on the differentially expressed CAlncRNA sequence set to screen out eight lncRNAs associated with OS (P < 0.01) (Fig. 3A). In order to prevent overfitting of the data and reduce errors, Lasso regression analysis was performed in this study (Fig. 3C,D). In addition, we constructed a risk score model based on a multifactorial Cox proportional risk regression analysis, with a score = MKLN1-AS × (1.08586410021725) + FOXD2-AS1 × (0.328542210980025) + LINC02870 × (0.42315377485594). The prognostic risk score for each patient were calculated based on the risk score model. The correlation heatmap showed the correlation of cuproptosis-related genes with the three lncRNAs of the model (Fig. 3B).

### 3.3 Prognostic value validation of the risk model

**Fig. 4:**
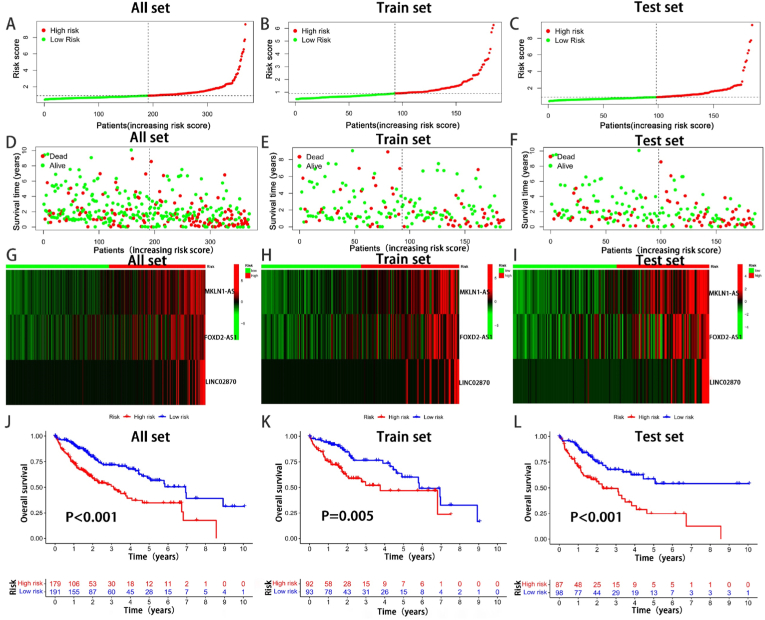
Validation of prognostic models for three CAlncRNAs. (A–I) Principal component analysis, risk score distribution, and survival status distribution for all, train, and test set. (J–L) Kaplan-Meier curves of all, train, and test set at different risk groups.

**Fig. 5:**
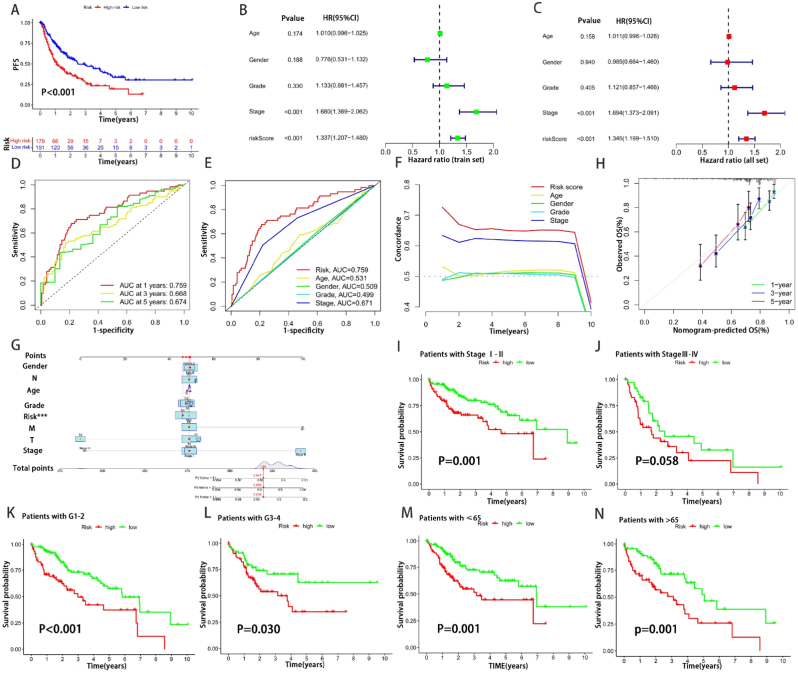
Prognostic value validation of the risk model. (A) K-M curves of OS differences stratified by TNM stage tumor grade, or age between the high- and low-risk groups in the TCGA all set. (B,C) Forest plot of clinical information and risk score associated with OS of the train and all set HCC patients based on univariate Cox regression analysis. (D) Time-dependent ROC curves to evaluate the accuracy of risk scores for predicting 1-year, 3-year, and 5-year survival of HCC samples. (E) ROC curves of the different clinical characteristics (age, gender, grade, stage) and risk score for multiple prognostic indicators of HCC samples. (F) C-index curve showing the accuracy of clinical characteristics and risk score in assessing HCC patients prognosis. (G) Nomogram constructed using the train set dataset. (H) Calibration curve for the nomogram model for predicting 1-, three- and 5 years OS. (I–N) K-M curves of OS differences stratified by TNM stage tumor grade, or age between the high- and low-risk groups in the TCGA all set.

To validate the prognostic performance of the model, patients in the validation group were divided into a high-risk group (n = 92) and a low-risk group (n = 92) based on the risk score formula. Specifically, the risk score from distribution Fig.s indicated the association between the higher score and lower the survival rate (Fig. 4A–F). Then the heatmap showed that all the three lncRNAs were highly expressed in the high-risk group (Fig. 4G–I). K-M survival analysis was performed, and the results showed HCC patients with high risk scores had significantly shorter survival times than low-risk patients (all:P<0.001, train:P<0.01 and test:P<0.001, Fig. 4J-L). Similarly, the PFS of high risk HCC patients was also shorter than low-risk patients significantly (**P<0.001**, Fig. 5A).

Subsequently, the train and all set clinical information and risk score were subjected to univariate and multivariate COX regression analyses with corresponding forest plots attached (Fig. 5B,C). The results showed that the p values of risk score and stage were less than 0.001 in both univariate and multivariate COX regression analyses, indicating that the risk score and stage constructed could be as independent prognostic factors. In addition, it was worth noting that the results of the forest plots showed lower HR values for our model compared to stage.

According to the risk model constructed in this study, the ROC curves for the 1-, 3- and 5-year survival rates of the train set were plotted with the AUC values 0.759, 0.668, and 0.674, respectively (Fig. 5D), indicating a high prognostic accuracy. The ROC curves of the different clinical characteristics (age, gender, grade, stage) and risk score showed the AUC of the risk score was the highest (Fig. 5E).

Similarly, the model constructed in this study had the largest concordance index in the C-index curve compared to other clinical characteristics, showing the advanced accuracy in assessing HCC patients prognosis (Fig. 5F).

A nomogram was built based on three independent prognostic factors, risk score, and stage (p < 0.001 both in univariate and multivariate COX), for predicting the 1, 3, and 5 year OS incidences of HCC patients (Fig. 5G). Next, we produced calibration plots for 1, 3, and 5 years and verified the accuracy of the nomogram in forecasting OS. The results validated that our prediction model has good prognostic accuracy at 1, 3 and 5 years (Fig. 5H). We next validated the applicability of the model in different clinical characteristics to better distinguish OS between high and low risk groups in patients with different stages, genders, and ages of HCC (Fig. 5I-N).

### 3.4 Molecular subtype analysis of different risk groups

**Fig. 6:**
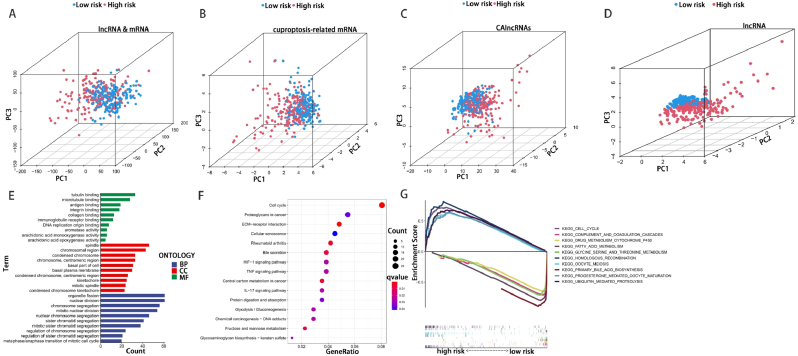
PCA, GO and KEGG analysis. Results of the PCA between low-risk and high-risk groups based on the dataset of whole-gene data (A), cuproptosis-related mRNA data (B), CAlncRNAs data (C), and lncRNAs data (D). (E) The top 30 GO biological processes of BP, CC, and MF. (F) The immune cell bubble of risk groups. (G) Gene set enrichment analysis of the top ten pathways significantly enriched in the risk groups.

We performed principal component analysis (PCA) to investigate the differentiation between the low-risk and high-risk groups based on the dataset of whole-gene data (mRNAs & IncRNAs), cuproptosis-related mRNA data, CAlncRNAs data, and lncRNAs data from the train set (Fig. 6A-D**).** The results showed that the IncRNAs data divided HCC into two more distinct parts in the train set, indicating the significant difference of cuproptosis status between the low-risk group and the high-risk group from HCC patients.

### 3.5 GO and KEGG analysis

GO and KEGG analysis of co-expressed mRNAs were performed to identify potential biological roles involved in the prognostic characterization of model. Results showed that the lncRNA co-expressed mRNAs were involved in 436 Biological Process (BP), 34 Molecular Function (MF), and 65 Cellular Component (CC). We showed the top ten entries of each of the three modules sorted by P value. GO analysis showed that these genes were significantly enriched in cancer-related networks, including organelle fission, nuclear division, spindle and tubulin binding (Fig. 6E). In addition, KEGG analysis showed that 11 of the top 15 pathways enriched in the high-risk group were highly correlated with tumor invasion, including the Cell cycle, ECM-receptor interaction and Central carbon metabolism in cancer pathways (all P < 0.01). The other four pathways were associated with immune function, such as IL-17 signaling pathway and TNF signaling pathway (**all P < 0.05**, Fig. 6F). Therefore, immunological analysis was enrolled for further illustrating for the model.

### 3.6 Immune statuses analysis of different risk groups

**Fig. 7:**
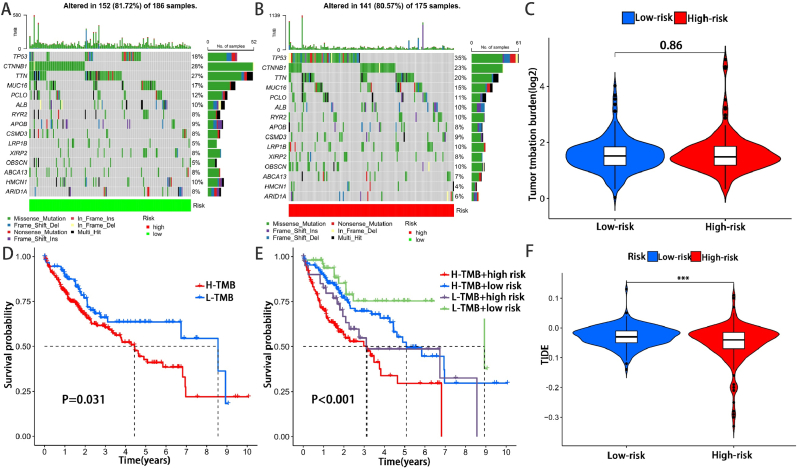
Immune statuses analysis of different risk groups. (A) Heatmap showing the immune related functions difference between high-risk and low-risk groups. (B) The correlation between risk score and TIICs analyzed by seven different quantification methods of immune infiltration estimations including TIMER, xCell, quanTIseq, MCP-counter, EPIC, CIBERSORT-ABS, and CIBERSORT. (C) ssGSEA algorithm showing the levels of infiltration of 16 immune cells in high-risk and low-risk groups. (D) Relationship between predictive features and 13 immune-related functions. (E) Expression of immune checkpoints in high-risk and low-risk groups. (F–K) The correlation between six significant checkpoint molecules and the risk scores of. *p< 0.05; **p< 0.01; ***p< 0.001.

The results of immune-related functions examination showed that immune-related functions were significantly different between high and low risk groups including type II IFN reponse, parainflammation and MHC class I (**P < 0.001**, Fig. 7D). Then, GSEA software was used to explore the high-risk group in the KEGG pathway in the entire set (Fig. S2). MultiGSEA result displayed that the top five pathways significantly enriched in the high-risk group were homologous recombination, cell cycle, ubiquitin mediated proteolysis, oocyte meiosis and progesterone mediated oocyte maturation. In contrast, drug metabolism cytochrome p450, complement and coagulation cascades, glycine serine and threonine metabolism, fatty acid metabolism and primary bile acid biosynthesis were markedly enriched in the low-risk group(**P < 0.001**, Fig. 6G). The heatmap of immune related functions showed that type II IFN response significantly enriched in low-risk HCC patients, however, MHC class I and parainflammation were conversely enriched in high-risk HCC patients (**all P < 0.001**, Fig. 7A). Immunocell correlation analyzed by seven software calculated bubble plots showed that several immune cells were associated with the high-risk group on different platforms such as neutrophil and macrophage at TIMER, CD4^+^ Th2 cell at XCELL, M1 macrophage at QUANTISEQ, monocyte at MCPCOUNTER, uncharacterized cell at EPIC, M0 macrophage at CIBERSORT-ABS (**all P < 0.001**, Fig. 7B,F–K). These findings were consistent with the difference result in ssGSEA of the immune functions (MHC class I, parainflammation and type II IFN response) and immune cells (aDCs, macrophage, mast cell, NK cell and Tregs) (Fig. 7C,D). Then we found that all immune checkpoint-associated genes were significantly overexpressed in high-risk patients, showed the advanced selection of appropriate immune checkpoint inhibitors for HCC risk model (Fig. 7E).

### 3.7 Tumor Mutational Burden and TIDE analysis

**Fig. 8:**
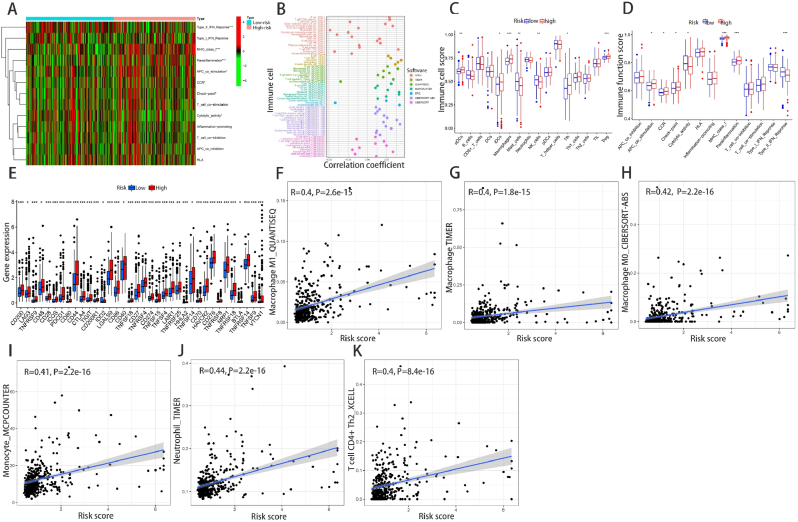
Tumor Mutational Burden and TIDE Analysis. (A–B) The top 20 genes’ TMB in the low-risk group and high-risk group. (C) Boxplot was used to visualize the TMB level between the low-risk group and high-risk group. (D) The survival difference between the high TMB group and low TMB group. (E)The survival status of patients with low or high risk-score in the high TMB group and low TMB group. (F) Boxplot was used to visualize the TIDE level between the low-risk group and high-risk group. ***p< 0.001.

We generated oncoplot based on the somatic mutation data of TGCA, and the results showed that the TMB score was higher in the high-risk group (Fig. 8A,B). However, no significance was observed in TMB between high and low risk group (Fig. 8C). Then, the samples were divided into low and high-mutation groups according to the TMB score. The high-mutation group had a significantly lower survival rate compared with the low-mutation group (**P < 0.05**, Fig. 8D). Further, the results showed that our model played an advance role in predicting more accurate survival rate than the TMB score (**P < 0.001**, Fig. 8E).

The TIDE algorithm is commonly used to assess the clinical outcome of patients on institute for Immune checkpoint inhibitors (ICI) therapy. A higher TIDE score may connected to greater likelihood of immune escape, more limited response to ICI, as well as the shorter survival time. The results of our TIDE analysis showed higher TIDE scores in patients with high-risk HCC (**P < 0.001**, Fig. 8F), suggesting that HCC patients in the high-risk group, to the extend, may not benefit from ICI therapy compared to the low-risk group.

### 3.8 Prediction of useful chemical drugs for the treatment of cellular component

**Fig. 9:**
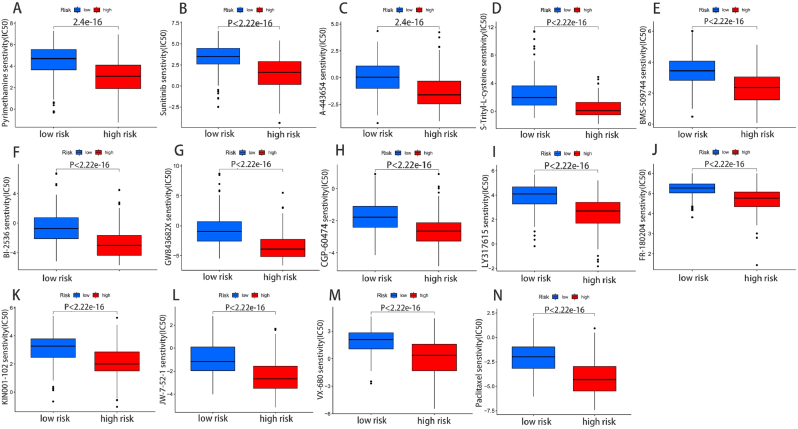
Prediction of useful chemical drugs for the treatment of cellular component. (A) Pyrimethamine, (B) Sunitinib, (C) A-443654, (D) S-Trityl-L-cysteine, (E) BMS-509744, (F) BI-2536, (G) GW843682X, (H) CGP-60474, (I) LY317615, (J) FR-180204, (K) KIN001-102, (L) JW-7-52-1, (M) VX-680, (N) Paclitaxel.

We used the “pRophetic” algorithm to calculate the IC50 value of 77 conventional low-risk and high-risk groups of chemotherapy drugs to assess the response of HCC patients to chemotherapy. The results showed a statistically significant difference in responses to almost all chemotherapy drugs between the two risk groups. Among the top 14 drugs, the high-risk group showed a higher sensitivity than those in the low-risk group (Fig. 9A-N).

### 3.9 Experimental verification of the three CAlncRNAs expression levels

**Fig. 10:**
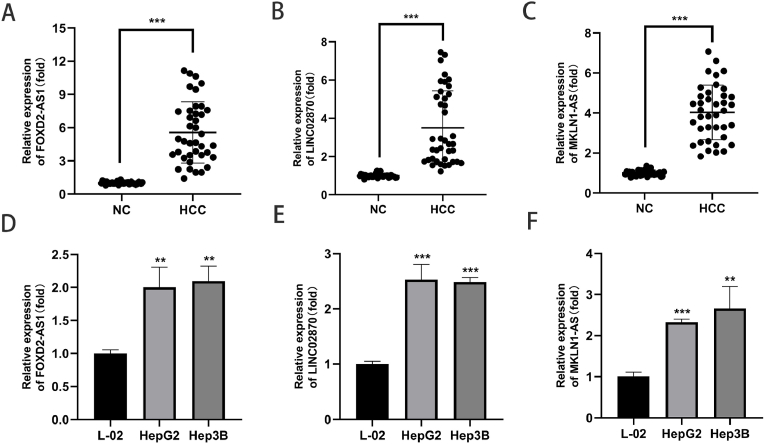
Expression level of the three CAlncRNAs in HCC tissues and cells. (A–C) Expression level of the three CAlncRNAs in paired HCC tumor (n = 39) or adjacent normal (n = 39) tissues by RT-qPCR. GAPDH was used as an internal control. (D–F) Expression level of the three CAlncRNAs in HepG2 and Hep3B by RT-qPCR. **p< 0.01; ***p< 0.001.

To further validate our findings, we measured the expression of the three CAlncRNAs (MKLN1-AS, FOXD2-AS1, LINC02870) by RT-qPCR in tumor and paracancer tissues from 39 HCC patients. The results showed that the expression levels of all three CAlncRNAs were significantly higher in HCC tumor tissues than the **levels** from paracancer **(P < 0.001**, Fig. 10A-C**)**. Next, the expression of the 3 CAlncRNAs in cell lines were measured (L02, HepG2, Hep3B). Similar to the tissue, the three CAlncRNAs were highly expressed in all HCC cell lines **(P < 0.01**, Fig. 10D-F**).** Our previous results indicated that all three CAlncRNAs were high-risk lncRNAs and PCR results simultaneously confirmed the reliability of the risk model.

### 3.10 Functional assays of the three CAlncRNAs

**Fig. 11:**
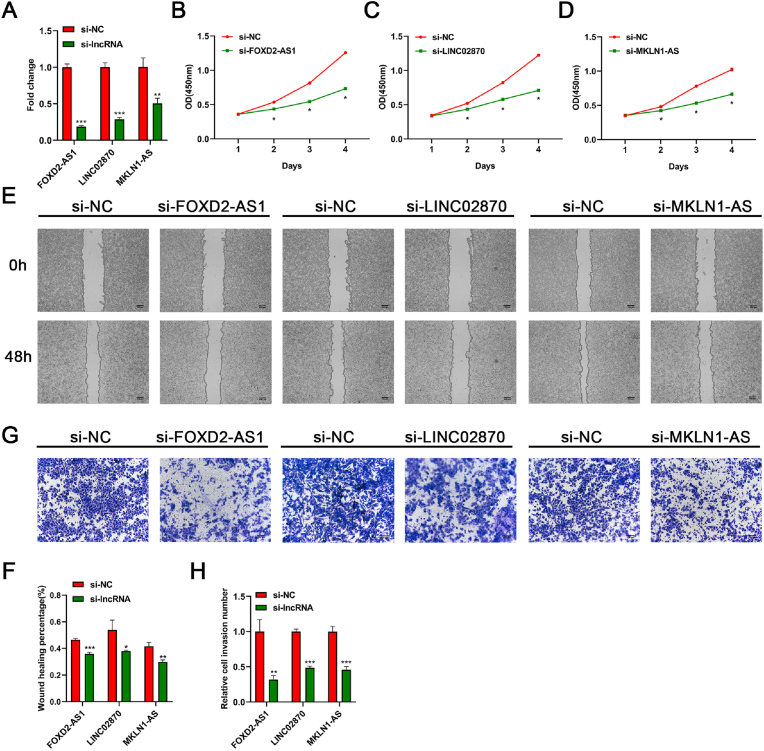
Functional assays of the three CAlncRNAs in HepG2 cells. (A) HepG2 cells were transfected with siRNA specifically targeting the three CAlncRNAs or si-NC, and siRNA-depletion efficiency was detected by RT-qPCR. GAPDH was used as an internal control. (B–D) The proliferation of HepG2 cells after transfection was detected by CCK-8 assays. (E, F) Confluent HepG2 cells were subjected to scratch wounding, and the pictures represented wound gaps at 0 h and 48 h. (G, H) The invaded HepG2 cells were representatively imaged. All results represent the average of three independent experiments ($\bar{x}$ ± SD). *p <0.05; **p < 0.01; ***p < 0.001.

HepG2 cell line was then selected for silencing of the three CAlncRNAs (MKLN1-AS, FOXD2-AS1, LINC02870) and the transfection efficiency were detected by qRT-PCR (Fig. 11A). The results of CCK8 revealed that the three CAlncRNAs silencing remarkably suppressed the proliferation of HepG2 cells (Fig. 11B–D). Moreover, wound healing assay showed that transfection of siRNAs significantly inhibited the migration in HepG2 cells, compared with the control groups (Fig. 11E, F). The invasion assay further indicated that the number of invaded cells was markedly reduced following silencing of the three CAlncRNAs in HepG2 cells (Fig. 11G, H). Furthermore, to specifically address the exact role of the three CAlncRNAs in apoptosis, flow cytometry analysis was performed in siRNAs-transfected HepG2 cells. As expected, the three CAlncRNAs silencing remarkably promoted apoptosis in HepG2 cells (Fig. 11). Collectively, these results suggested the three CAlncRNAs exert tumor-promoting activities.

## 4 Discussion

MKLN1-AS is a good potential diagnostic and prognostic biomarker and therapeutic target for HCC. Studies have shown that MKLN1-AS acts as a miR-654-3p sponge competing with HDGF to play a pro-cancer role in liver cancer progression [[24], [25], [26]]. FOXD2-AS1 was significantly upregulated in HCC cells compared with LO2, and the knockdown of FOXD2-AS1 inhibited HCC cell proliferation, enhanced apoptosis, and suppressed cell invasion and migration [27]. Sui et al. [28]showed that knockdown of FOXD2-AS1 decreased TMEM9 expression and increased the sensitivity of HepG2 and Huh7 cells to sorafenib. Chang et al. [29] found that high expression of FOXD2-AS1 was significantly associated with lower OS in HCC patients. However, Ren et al. [30] reported that FOXD2-AS1 was not associated with OS or DFSin cutaneous melanoma. This difference should be due to the heterogeneity of tumors and the biological functions of lncRNAs which can modify the biological behavior of different tumors in different directions [31]. In addition, study showed that the risk score involving LINC02870 was an independent prognostic predictor for HCC patients, superior to traditional clinicopathological factors by multivariate analysis [32]. Currently, there is only one study on LINC02870, and the exploration of such newly discovered lncRNAs can shed a light to elucidate the pathogenesis of HCC and suggest novel approaches for tumor targeting therapy. Altogether, our CAlncRNAs (MKLN1-AS [25], FOXD2-AS1 [33], LINC02870 [32]) were all identified as high-risk lncRNAs, which is consistent with previous studies.

In contrast to previous studies using only online tools [34], our study employed the "edgeR" package in the R language to analyze the latest RNA-seq data from TCGA. In addition, to prevent overfitting of the model, we used multi-factor Cox regression combined with LASSO regression analysis for further screening of variables. K-M survival analysis and ROC curve analysis showed that our model had a significant effect on the prognosis of HCC patients. The AUC value of ROC curve showed the model had acceptable prediction accuracy (AUC = 0.750, 0.668 and 0.674 in 1, 3 and 5 years respectively). It is worth emphasizing that the AUC of 0.759 for our risk score was more accurate than other clinical factors and models in other literatures [35,36] in predicting the survival of HCC patients.

In addition, the repeatability and accuracy of the prognostic model were confirmed by the validation analysis of the test set and the all set. The risk score of the model had independent prognostic significance for HCC patients was determined by univariate and multivariate Cox regression analysis. The PCA analysis showed that CAlncRNAs could effectively stratify HCC patients and these stratified HCC patients exhibited clear intrinsic biological traits.

We sought to assess the precise mechanism of action of copper overload from the viewpoint of cuproptosis, which plays a regulatory function in several cancer stages, including HCC. GO analysis suggested that the function of cuproptosis may be related to organelle fission and nuclear division while both KEGG and GSEA analyses indicated that cuproptosis was primarily engaged in the cell cycle pathway. Therefore, we hypothesized that cuproptosis affects the prognosis of HCC patients mainly by regulating cell proliferation. Increasingly, studies [37,38] have shown that lncRNAs are critical gatekeeper molecules in the cell cycle process, during which dysregulation of lncRNAs can lead to immortalization of cancer cells. Xu et al. [39] by silencing FOXD2-AS1 arrested HCC cell cycle in the G0/G1 phase and inhibit colony formation, cell proliferation, and subcutaneous tumor growth *in vivo*. Zhao et al. [40] constructed a prognostic model of lncRNAs, including MKLN1-AS, for predicting HCC with enriched functions in cell cycle-related biological processes or pathways, which were consistent with our findings. We also noted that other cuproptosis -related risk models have been reported in HCC [41]. Compared to this study, our model has some advantages. First, not only do we validate the predictive value of the model separately in more detailed clinical subgroups, but our model have higher AUC values in 1, 3, and 5. Secondly, we also performed an innovative PCA analysis and confirmed that our model can well differentiate between high and low risk groups of patients. In addition, our model incorporated only three genes, meaning that it may be easier to apply in clinical practice.

In recent years, immunotherapy, represented by ICI such as programmed-cell death 1 pathway (PDCD1/L1), has emerged as a crucial new therapeutic option for HCC treatment [42]. Our ssGSEA differential analysis showed that some immune cells (Macrophages, Treg) and immune functions (MHC class I, Parainflammation) were abnormally active in the high-risk group for HCC. Tregs, a subpopulation of CD4^+^ T cells, which are frequently linked to a poor prognosis for HCC, directly promote tumor escape through a variety of contact-dependent and contact-independent mechanisms and severely suppress immunological responses [43]. Zhu et al. [44] found that tumour-associated macrophage (TAMs) and tumor immune escape were closely related, and the efficacy of immune checkpoint inhibitors in HCC was improved by inhibiting the migration of TAMs. Furthermore, establishing predictive biomarkers for checkpoint immunotherapy is essential to maximize the benefits of treatment [45]. Although these ICI monotherapy treatments showed a survival benefit in some patients, the response was suboptimal and may be attributable to strong immunosuppressive TME in the liver. Immune checkpoint analyses revealed that the high-risk group had considerably higher expression levels of all checkpoints than the low-risk group. Given the ICI expression levels is positively correlated to patient survival and response to immune checkpoint blockade (ICB) therapy [46], we screened for several of those checkpoints with the most significant differences (PDCD1, CTLA-4, CD200, TNFRSF9, CD28, ICOS, CD80, CD44). Currently, the most studied checkpoint proteins *in vitro* and with the highest clinical relevance are cytotoxic T lymphocyte–associated antigen 4 (CTLA-4), programmed cell death protein-1 (PDCD1), and programmed cell death ligand 1 (PD-L1) for HCC patients [47]. Studies have shown that Tregs constitutively express CTLA-4 to exert its immunosuppressive, and Treg-specific CTLA-4 deficiency has been shown to interfere with immune self-tolerance and suppressive function of Treg *in vivo* and promote tumor immunity [48,49]. Overall, immune checkpoint expression may be dysregulated in the HCC microenvironment, and cancer immunotherapy based on CTLA-4, PDCD1 and PD-L1 inhibitors may achieve antitumor therapeutic effects by improving Treg cell-mediated immune responses.

The TIDE score combines the expression characteristics of T-cell dysfunction and T-cell exclusion to assess the probability of tumor immune escape and to predict ICI treatment efficacy [50]. TIDE has the advantage of being able to predict the clinical response of ICB based on pre-treatment tumor characteristics [50]. Our study showed that patients in the low-risk subgroup had significantly higher TIDE scores, suggesting that patients in the high-risk group with HCC are less likely to experience immune escape and may respond better to ICI. In fact, a higher TMB implies a better response rate to ICI therapy for cancer. We found higher TBM in the high-risk group and less survival time in the high-risk TBM group compared to the low-risk group. Noted that in the differences in TBM between high and low risk groups, statistical significance is at a marginal level (P = 0.86), which may be due to the limited sample size. Finally we found two most sensitive compounds (A-443654 & Pyrimethamine) in patients with high-risk HCC. Compound A-443654 interferes with tumor cell mitotic progression and bipolar spindle formation by specifically inhibiting Akt [51]. And pyrimethamine is considered to be the greatest HCC-specific cytotoxicity drug through image-based phenotypic screening in co-cultures of HCC cells with hepatocytes [52]. In summary, HCC patients in the high-risk group have a better ICB response and A-443654 & Pyrimethamine are the most promising candidates.

In our molecular functional experiments, we found the expression of MKLN1-AS, FOXD2-AS1 and LINC02870 were significantly upregulated in HCC and exerted tumor-promoting activities, which were consistent with other’s. 33000222,36583796,32558350 In addition, we also find that silencing the three CAlncRNAs could promote HCC cells apoptosis. Actually, the mechanism of cell death induced by copper ion carriers is different from the apoptotic cell death pathway.35298263 Therefore, we hypothesize that there may be a mechanism by which these CAlncRNAs link cuproptosis and apoptosis to work together to regulate HCC cell life cycle.

Only three lncRNAs are included in our model, which lowers the expense of patient testing as well as the burden on clinicians. However, there are still many shortcomings. First, the TCGA database, whose samples were mostly made up of Caucasians and African-Americans, served as the source for all the data used in this study, whereas the specimens we collected were from Asians, it remains to be further verified whether there are differences in expression levels between the ethnic groups. Second, our model only incorporated predictive survival data of patients and did not incorporate additional clinicopathological information and other risk factors properly comprehend the importance of these risk variables.

Based on the aforementioned study and other studies, our developed CAlncRNAs-based risk score model has a good predictive value and the capacity to predict the response to immunotherapy in HCC patients. In addition, our study suggested that CAlncRNAs features and their potential mechanisms to enhance the understanding of cuproptosis in TME cell infiltration and immune evasion and offer fresh perspectives to guide more effective immunotherapeutic strategies.

## Author contributions

Zhu Liangyu: Drafting the manuscript. Zhang Bochao: Analysis and interpretation of data. Yin Guoquan: Acquisition of data. Zhang Yuan: Verification. Li Heng: Revising the manuscript critically for important intellectual content. Zhou Hanyu: Oversight and leadership responsibility for the research activity planning and execution, including mentorship external to the core team.

## Funding

This work was supported by grants from the Youth Project Training Fund of Wannan Medical College [grant no. WK2020ZF01], Wannan Medical College Yijishan Hospital introduced talent research fund [grant no. YR202102], "Applied Basic Research Project" of Wuhu Science and Technology Project [grant no. 2022jc71].

## Publisher’s note

All claims expressed in this article are solely those of the authors and do not necessarily represent those of their affiliated organizations, or those of the publisher, the editors and the reviewers. Any product that may be evaluated in this article, or claim that may be made by its manufacturer, is not guaranteed or endorsed by the publisher.

## Declaration of competing interest

The authors declare that the research was conducted in the absence of any commercial or financial relationships that could be construed as a potential conflict of interest.

## Data Availability

Data will be made available on request.
