# Supplementary material for: Bioinformatics prediction and experimental verification identify cuproptosis-related lncRNA as prognosis biomarkers of hepatocellular carcinoma
Source: Biochem Biophys Rep. 2023 Jun 21;35:101502. doi: 10.1016/j.bbrep.2023.101502 (PMC10322676; doi:10.1016/j.bbrep.2023.101502)
Supplement: Multimedia component 1 [file mmc1.docx]

**The citation for supplementary materials was highlighted as red in the following text. The Fig S2 was corrected into Fig.6G. Please check the line 92 in this file. In addition, we also re-marked all the supplementary tables with red color. Please check the line 19, 29, 47, 85 in this file.**

**2. Materials and methods**

**2.7 TMB, TIDE and Immune correlation analysis**

First, the somatic mutation data (TCGA.BRCA.varscan.DR-10.0.somatic) was downloaded from TCGA. Then the “maftools”, “survival” and “survminer” R packages were used to integrate the data and analyze the diverse survival of TMB between high and low risk group. The TIDE score between two groups was downloaded from online database (http://tide.dfci.harvard.edu/).

The immunocyte infiltration analysis was performed between high and low risk groups according to the Tumor Immune Estimation Resource (TIMER) 2.0 (<http://timer.cistrome.org/>), with the infiltration fraction calculated in TME via Wilcoxon signed-rank test, “limma”, “scales”, “ggplot2” and “ggtext” R packages.

To explore the Potential immune checkpoint, we compared 47 immune checkpoint (**Table.S1**) related gene expression between high and low risk groups by “limma”, “reshape2”, “ggplot2” and “ggpubr” R packages.

In addition, immune-related function heatmap was performed according to the immune function set file (**immune.gmt**) by “limma”, “GSVA”, “GSEABase”, “pheatmap” and “reshape2” R packages between the two groups.

**3. Results**

**3.1 Identification of Differentially Expressed CAlncRNAs**

We downloaded 50 normal samples and 374 HCC samples from The Cancer Genome Atlas (TCGA) matrix. According to the expression of 19 cuproptosis genes, we screened 509 CAlncRNAs (**Table.S2**) with the standard of correlation coefficients > 0.4 and P < 0.001. Among the CAlncRNAs, we got 254 differentially expressed CAlncRNAs (|Log_2_FC| > 1 and P < 0.05) between the normal and tumor groups, including eight downregulated lncRNAs and 246 upregulated lncRNAs. All these genes were clustered and analyzed by the relevant volcano and heatmaps (**Fig. 2A,B**). The potential correlations between the lncRNAs and mRNA were demonstrated by network Fig., sankey relational diagram and PPI.(**Fig. 2C-E**).

**3.2 Construction the Prognostic risk assessment model with CAlncRNAs**

Using random grouping method, the HCC differentially expressed CAlncRNAs data were equally divided into train and test sets. Combined with clinical information, univariate COX regression analysis was performed on the differentially expressed CAlncRNA sequence set to screen out eight lncRNAs associated with OS (P < 0.01) (**Fig. 3A**). In order to prevent overfitting of the data and reduce errors, Lasso regression analysis was performed in this study (**Fig.3 C,D**). In addition, we constructed a risk score model based on a multifactorial Cox proportional risk regression analysis, with a score = MKLN1-AS × (1.08586410021725) + FOXD2-AS1 × (0.328542210980025) + LINC02870 × (0.42315377485594). The prognostic risk score for each patient were calculated based on the risk score model **(Table.S3)**. The correlation heatmap showed the correlation of cuproptosis-related genes with the three lncRNAs of the model (**Fig.3B**).

**3.3 Prognostic Value validation of the Risk Model**

To validate the prognostic performance of the model, patients in the validation group were divided into a high-risk group (n = 92) and a low-risk group (n = 92) based on the risk score formula. Specifically, the risk score from distribution Fig.s indicated the association between the higher score and lower the survival rate (**Fig. 4A-F**). Then the heatmap showed that all the three lncRNAs were highly expressed in the high-risk group (**Fig. 4G-I**). K-M survival analysis was performed, and the results showed HCC patients with high risk scores had significantly shorter survival times than low-risk patients (**all:P<0.001, train:P<0.01 and test:P<0.001, Fig. 4J-L**). Similarly, the PFS of high risk HCC patients was also shorter than low-risk patients significantly (**P<0.001, Fig. 5A**).

Subsequently, the train and all set clinical information and risk score were subjected to univariate and multivariate COX regression analyses with corresponding forest plots attached (**Fig. 5B,C**). The results showed that the p values of risk score and stage were less than 0.001 in both univariate and multivariate COX regression analyses, indicating that the risk score and stage constructed could be as independent prognostic factors. In addition, it was worth noting that the results of the forest plots showed lower HR values for our model compared to stage.

According to the risk model constructed in this study, the ROC curves for the 1-, 3- and 5-year survival rates of the train set were plotted with the AUC values 0.759, 0.668, and 0.674, respectively (**Fig. 5D**), indicating a high prognostic accuracy. The ROC curves of the different clinical characteristics (age, gender, grade, stage) and risk score showed the AUC of the risk score was the highest (**Fig. 5E**).

Similarly, the model constructed in this study had the largest concordance index in the C-index curve compared to other clinical characteristics, showing the advanced accuracy in assessing HCC patients prognosis (**Fig. 5F**).

A nomogram was built based on three independent prognostic factors, risk score, and stage (p < 0.001 both in univariate and multivariate COX), for predicting the 1, 3, and 5 year OS incidences of HCC patients (**Fig. 5G**). Next, we produced calibration plots for 1, 3, and 5 years and verified the accuracy of the nomogram in forecasting OS. The results validated that our prediction model has good prognostic accuracy at 1, 3 and 5 years (**Fig. 5H**). We next validated the applicability of the model in different clinical characteristics to better distinguish OS between high and low risk groups in patients with different stages, genders, and ages of HCC (**Fig. 5I-N**). And the differences between clinical characteristics and sets were listed in **Table S4**.

**3.6 Immune Statuses Analysis of different risk groups**

The results of immune-related functions examination showed that immune-related functions were significantly different between high and low risk groups including type II IFN reponse, parainflammation and MHC class I (**P < 0.001, Fig. 7D**). Then, GSEA software was used to explore the high-risk group in the KEGG pathway in the entire set (**Fig.6G**). MultiGSEA result displayed that the top five pathways significantly enriched in the high-risk group were homologous recombination, cell cycle, ubiquitin mediated proteolysis, oocyte meiosis and progesterone mediated oocyte maturation. In contrast, drug metabolism cytochrome p450, complement and coagulation cascades, glycine serine and threonine metabolism, fatty acid metabolism and primary bile acid biosynthesis were markedly enriched in the low-risk group (**P < 0.001, Fig. 6G**). The heatmap of immune related functions showed that type II IFN response significantly enriched in low-risk HCC patients, however, MHC class I and parainflammation were conversely enriched in high-risk HCC patients (**all P < 0.001, Fig. 7A**). Immunocell correlation analyzed by seven software calculated bubble plots showed that several immune cells were associated with the high-risk group on different platforms such as neutrophil and macrophage at TIMER, CD4^+^ Th2 cell at XCELL, M1 macrophage at QUANTISEQ, monocyte at MCPCOUNTER, uncharacterized cell at EPIC, M0 macrophage at CIBERSORT-ABS (**all P < 0.001, Fig. 7B,F-K**). These findings were consistent with the difference result in ssGSEA of the immune functions (MHC class I, parainflammation and type II IFN response) and immune cells (aDCs, macrophage, mast cell, NK cell and Tregs) (**Fig. 7C,D**). Then we found that all immune checkpoint-associated genes were significantly overexpressed in high-risk patients, showed the advanced selection of appropriate immune checkpoint inhibitors for HCC risk model (**Fig. 7E**).
